# Development of Xanthan Gum-Modified Coal-Fly-Ash-Based Cementitious Firefighting Materials with Improved High-Temperature Resistance for Coal Mines

**DOI:** 10.3390/ma18184246

**Published:** 2025-09-10

**Authors:** Guolan Dou, Peng Chen, Menghan Wang, Jingyu Wang, Xiaoxing Zhong, Shuangming Wei

**Affiliations:** 1 Key Laboratory of Gas and Fire Control for Coal Mines, China University of Mining and Technology, Xuzhou 221116, China; 2 State Key Laboratory of Coal Mine Disaster Prevention and Control, China University of Mining and Technology, Xuzhou 221116, China; 3 School of Safety Engineering, China University of Mining & Technology, Xuzhou 221116, China; 4 Xinjiang Key Laboratory of Coal Mine Disaster Intelligent Prevention and Emergency Response, Xinjiang Institute of Engineering, Urumqi 830023, China

**Keywords:** coal fly ash, xanthan gum, crosslink, high-temperature resistance, cementitious firefighting material

## Abstract

In this study, xanthan gum (XG)-modified coal-fly-ash-based cementitious materials were synthesized to realize the resource utilization of coal fly ash and to develop a low-carbon emission cementitious sealing material that can substitute cement-based sealing material to prevent coal fires. The optimal formulation for coal-fly-ash-based mining cementitious sealing material was developed using response surface methodology based on Box–Behnken Design. The optimized formulation was obtained with a coal fly ash-to-precursor ratio of 0.65, alkali-activator modulus of 1.4, and alkali-activator dosage of 7.5%. Under the optimal conditions, the initial and final setting time were 26 min and 31 min, respectively, fluidity was 245 mm, and the 7-day compressive strength approached 36.60 MPa, but there were still thermal shrinkage and cracking phenomena after heating. XG was then introduced to improve the thermal shrinkage and cracking of coal-fly-ash-based cementitious materials. Incorporating 1 wt.‰ XG was found to decrease the fluidity while maintaining the setting time and increasing the 1-day and 7-day compressive strength by 15.44% and 1.97%, respectively. The results demonstrated that the gels generated by XG cross-linking and coordinating with Al^3+^/Ca^2+^ were interspersed in the original C(N)-A-S-H gel network, which not only made the 1 wt.‰ XG modified coal-fly-ash-based cementitious material show minor expansion at ambient temperatures, but also improved the residual compressive strength, thermal shrinkage resistance and cracking resistance in comparison to unmodified cementitious material. However, due to the viscosity of XG and the coordination of Al^3+^ and non-terminal carboxyl groups in XG breaking the gel network, XG incorporation should not exceed 1 wt.‰ as the compressive strength and fluidity are decreased.

## 1. Introduction

Coal is one of the primary global energy sources, and its position as the main energy in China will remain unchanged in the future for a long time [1,2,3,4]. According to statistics, coal production exceeded 4.7 billion tons in 2023 in China [5]. However, coal fire disasters have always hampered coal mining and storage. Coal fire not only causes a great waste of fossil fuels, but also generates a large number of toxic and hazardous gases [6,7]. Coal spontaneous combustion may also result in gas and coal dust explosion. Therefore, coal fire has emerged as a great challenge that must be addressed to ensure the safety of coal mining.

One of the most effective methods to prevent coal fire is to seal air leakage channels and reduce oxygen supply to the fire area [8,9]. At present, the commonly used sealing materials in coal mines are organic and inorganic sealing materials. Among them, organic sealing materials, which primarily consist of organic polymers, can rapidly expand and solidify to seal the leakage air. These materials are classified mostly as polyurethane foam [10,11], phenolic foam [12,13] and urea formaldehyde foam [14,15]. However, organic sealing materials are usually expensive, and there are safety issues in underground applications, making them unsuitable for large-scale sealing in coal mines. Yellow mud slurry and cement-based materials are the most common inorganic sealing materials [16,17]. However, yellow mud slurry often fails to accumulate effectively in regions with large cracks and air leakage due to the “trench” effect, while cement-based materials, though simple to prepare and use, are associated with high energy consumption and significant carbon emissions during production [18,19]. These environmental impacts conflict with the strategic goals of carbon peak and neutrality, underscoring the need for more sustainable alternatives to conventional cement-based sealing materials.

It has been reported that substituting cement with low-carbon supplementary cementitious material is an effective method to decrease carbon dioxide emissions. Coal fly ash, a by-product of coal-fired power plants, has been used to replace cement [20,21]. However, coal fly ash is less reactive than cement, which may lead to delayed setting times and reduced early strength development, especially when used in large quantities [22,23]. Despite this limitation, coal-fired power plants provide more than 70% of China’s electricity, resulting in a massive accumulation of coal fly ash in power plants each year [24]. The large-scale disposal of coal fly ash poses significant environmental pollution risks and consumes substantial land resources. Therefore, there is an increasing demand for effective strategies to utilize coal fly ash as a viable alternative to cement.

Recently, alkali-activated aluminosilicate industrial wastes exhibiting cement-like properties and low CO_2_ emissions have been investigated as a potential alternative to cement [25,26,27]. Coal fly ash, which is mostly composed of silicon dioxide (SiO_2_), aluminum oxide (Al_2_O_3_) and calcium oxide (CaO), has been widely used as the precursor for this alkali-activated material [28,29]. Many investigators have used various alkaline activators including NaOH [30], Na_2_CO_3_ [31], and NaOH/waterglass with varied SiO_2_/Na_2_O ratios [32,33] to activate fly ash. However, Class F fly ash with a CaO content less than 10% shows poor reactivity, resulting in slow reaction kinetics and extended setting times [34]. In order to improve the reactivity, blast furnace slag was added to the blend with fly ash. Puertas et al. studied the strength behavior of alkali-activated fly ash and blast furnace slag blends and discovered that the 28-day compressive strength of the 50% fly ash/50% slag mixture activated with 10 M NaOH was about 50 MPa [28]. Marjanović compared the reactivity of FA in the reaction of alkali activation improved by the mechanical activation of FA and by blending FA with blast furnace slag, and found that a highly cross-linked structure, which probably consisted of N-A-S-H and C-A-S-H in an alkali-activated FA and blast furnace slag mixture, increased the compressive strength [35]. Although studies have investigated the effect of the FA/blast furnace slag ratio, the alkali activator composition and the concentration of NaOH on setting behavior [36,37,38], the systematic optimization of these parameters remains limited. Therefore, one goal of this paper is to employ response surface methodology for optimizing the mix design of alkali-activated fly ash and a blast furnace slag blend, aiming to develop a coal-fly-ash-based cementitious firefighting material with a stable structure and appropriate setting time.

Additionally, the alkali-activated cementitious materials generated from fly ash and other industrial solid wastes still exhibit cracking and shrinkage, particularly after exposure to heat [39,40], which negatively affects their air-sealing performance. Therefore, improving the cracking and shrinkage resistance of fly-ash-based cementitious materials is another key issue in developing fly-ash-based cementitious sealing materials. Previous studies have shown that organic polymers rich in proton-donating groups such as sodium alginate and lignin can improve the structural stability and thermal mechanical properties of alkali-activated materials [41,42]. Similarly, Liu et al. also studied the water bleeding and viscosity of cement grout doped with various polysaccharide at high temperatures, and they discovered that active functional groups within the polysaccharides crosslinked with Ca^2+^ and Al^3+^ in cement grout to form networks [43]. Xanthan gum (XG) is a non-toxic anionic polysaccharide that consists of repeating units of glucose, mannose, and glucuronic acid linked with *β* glycoside, and contains carboxyl and hydroxyl groups in its structure. The carboxyl groups in the backbone of XG have been reported to interact with Ca^2+^ to generate a crosslinked macromolecular network during reverse osmosis fouling, thus forming a gel layer [44]. Xanthan gels can also be formed by combination with Al^3+^. For example, Malti et al. investigated sodium carboxymethyl xanthan gum crosslinking with Al^3+^ [45]. Rodd et al. studied the gelation behavior of xanthan-Al(III) gels [46]. XG has also been shown to improve the rheological properties of geopolymer pastes used in 3D-pringting application [47]. Given that Ca^2+^ and Al^3+^ were generated during the preparation of alkali-activated materials [43], XG is expected to interact with these ions to form crosslinked structures within the FA-based matrix, thereby improving the cracking and shrinkage resistance performance.

In light of these considerations, the present research aims to develop an optimized, environmentally friendly XG-modified FA-based mining cementitious material for fire prevention. The study emphasizes the optimization of the blast furnace slag/FA ratio, as well as the dosage and modulus of the alkali-activator to create a stable structure, and further investigates the role of XG in forming a crosslinked network with Ca^2+^ and Al^3+^ to improve the heat resistance, cracking and shrinkage resistance properties. It is worth noting that several previous studies have explored the development of eco-friendly construction materials incorporating recycled components [48,49]. However, the combination of response surface optimization and polysaccharide-based modification for mining sealing materials represents a novel approach that distinguishes this work from the existing literature.

## 2. Experimental

### 2.1. Raw Materials

The precursors used in this study were coal fly ash (FA) and ground granulated blast furnace slag (GBFS) supplied by Kexu Building Material Co., Ltd., Shijiazhuang, Hebei Province, China and Yixiang Materials Co., Ltd., Sanmenxia, Henan Province, China, respectively. The main composition of metal oxides in FA and GBFS was determined by X-ray fluorescence analysis (XRF) (S8 Tiger, Bruker, Billerica, MA, USA), as shown in Table 1. FA, which is categorized as class F fly ash by ASTM C618, is primarily composed of SiO_2_ and Al_2_O_3_, containing only 2.3% CaO. Meanwhile, CaO, SiO_2_, and Al_2_O_3_ are the main constituents of GBFS, with a CaO value of 36.8%.

**Figure 1:**
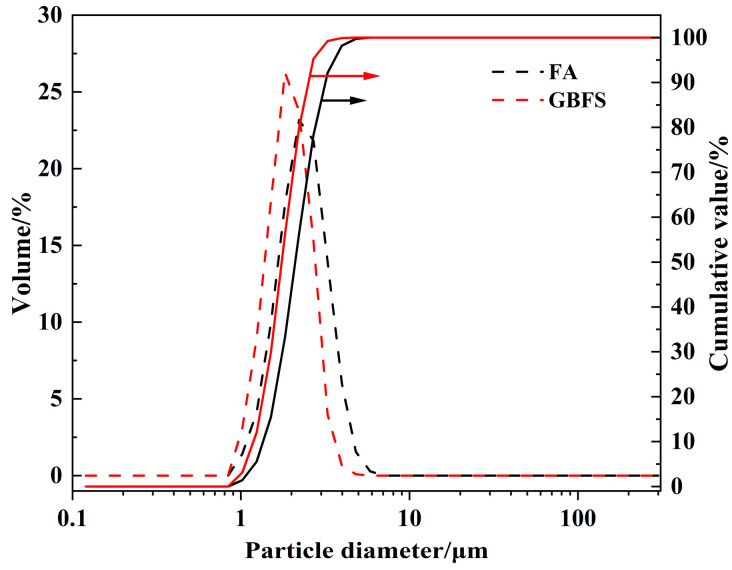
The particle size distribution curve of FA and GBFS (Black and red solid lines represent cumulative value for FA and GBFS, respectively).

The particle size distributions of FA and GBFS were measured by laser diffraction (Weina, Jinan Province, China) and the results are shown in Figure 1. The d10, d50, and d90 of FA and GBFS are 1.36 µm and 1.19 µm, 2.11 µm and 1.76 µm, 3.19 µm and 2.53 µm, respectively, indicating that GBFS has finer particles compared to FA.

In this paper, FA-based cementitious firefighting materials were prepared by alkali-activation, with the alkali-activator consisting of sodium silicate (Na_2_SiO_3_) and sodium hydroxide (NaOH). The Na_2_SiO_3_ solution with a solid composition of 26.98% SiO_2_ and 8.53% Na_2_O with a modulus of 3.3 was provided by a local medical station. NaOH solution (10 M) was prepared by dissolving sodium hydroxide pellets (MACHLIN reagent Co., Ltd., Shanghai, China) in tap water.

Moreover, xanthan gum (XG) was introduced to modify the cementitious material, and the XG utilized was of food grade and obtained from a nearby medical station.

### 2.2. Preparation and Curing of FA-Based Cementitious Material

**Figure 2:**
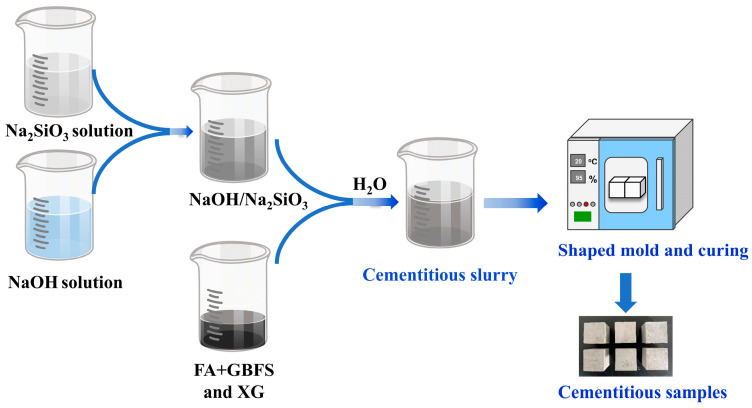
Preparation of FA-based cementitious materials [42].

The schematic of the procedure for preparing FA-based cementitious materials is shown in Figure 2. Prior to mixing, a 10 M NaOH solution was blended with a Na_2_SiO_3_ solution. The resulting alkaline activator was sealed and aged at room temperature for 24 h. The solid precursors (FA and GBFS), as well as the modifier (XG), were then dry mixed using a mechanical mixer at 120 rm for 2 min to achieve a homogeneous powder blend. The pre-prepared alkaline solution was then gradually added to the dry mixture over a period of 2 min under continuous mixing. Additional water was introduced to adjust the water-to-precursor ratio to the prescribed value. The mixing process continued for another 5 min at 200 rpm to ensure a consistent and workable slurry. The homogeneous slurry was subsequently poured into 20 mm × 20 mm × 20 mm cubic molds and vibrated for 60 s to remove entrapped air. All samples were stored in a standard curing chamber maintained at 95% relative humidity and a temperature of 20 ± 2 °C for 24 h. After demolding, the specimens were returned to the same curing environment and allowed to remain until reaching the designated testing ages. Preliminary tests were conducted to optimize the base cementitious material without XG, which served as the reference and was labeled as FG. Subsequent main tests involved the incorporation of XG as a modifier, with the XG-modified specimens designated as XFG.

### 2.3. Experimental Design

Since the FA-based cementitious slurry is a multi-component system, the precursor composition, the dosage of the alkali-activator, the modulus of the alkali-activator and the water-to-precursor ratio all affect the structure and properties of the cementitious material. By modeling the data, response surface analysis can not only determine the independent variables that play a major role in multiple factors and determine the interaction between variables, but can also select the optimal conditions of variables with the allowed expected value. To establish optimum experimental conditions for FA-based cementitious material, a three-factor and three-level Box–Behnken design was developed using the response surface methodology under a fixed water-to-precursor ratio of 0.5. Table 2 displays the factors considered in the cementitious material design. Factor 1 is the precursor composition, and in this study, the GBFS-to-precursors (FA + GBFS) ratio is used. Factor 2 is the dosage of the alkali-activator, the percentage of Na_2_O in the alkali-activator relative to the total amount of precursors is used to describe the activator dosage, and factor 3 is the modulus of the alkali-activator, which is the molar ratio of SiO_2_ to Na_2_O.

Preliminary tests focused on optimizing the cementitious material without XG through the response surface analysis. The response variables, including the 1-day compressive strength, 3-day compressive strength and 7-day compressive strength, were involved to model the response surface model for response variables. The focus on these short-term compressive strength properties is due to the intended application of the material in rapidly sealing the air leakage channels in coal mines to prevent the spontaneous combustion of coal. In such underground environments, where surrounding rock stresses and dynamic disturbances may manifest shortly following material injection, early-age mechanical performance is critical to ensure effective and timely sealing, providing resistance to deformation and airflow pressure. Analysis of variance (ANOVA) was performed using Design-Expert 12 software on the data for each response variable, with a 95% confidence interval to identify the terms of the factors that significantly influenced the 1-day, 3-day and 7-day compressive strengths and obtain the optimal formulation of FA-based cementitious material.

Based on the experimental results, the following second-degree polynomial model can be developed to describe the increases in the compressive strength of the cementitious material as a function of the three independent components (Equation (1)).

(1) $$Y=\beta_{0}+\sum_{i=1}^{3}\beta_{i}X_{i}+\sum_{i=1}^{3}\beta_{ii}X_{i}^{2}+\sum_{i=1}^{3}\sum_{j=i+1}^{3}\beta_{ij}X_{i}X_{j}$$

In this equation, *Y* represents the response value, *β*_0_ is the constant coefficient, *β*_i_, *β*_ii_, *β*_ij_ are the linear regression coefficient, quadratic coefficient, and interactive coefficient, respectively. *X*_i_ and *X*_j_ are factors that act independently.

Main tests with XG: Based on the optimal ingredients of the FA-based cementitious material identified from the preliminary tests, XG was incorporated into the precursor blend at concentrations of 0.1%, 0.2% and 0.3% of the total precursor mass. The resulting XG-modified mixtures, designated as XFG, were subsequently evaluated for key performance properties.

### 2.4. Test Methods

The compressive strength of the specimen after curing was measured using a microcomputer-controlled universal material tester (YAW4206, WANCE, Shenzhen, China) in accordance with Chinese GB/T 17671-1999 (Method of testing cements-Determination of strength) [50]. The key parameters included a displacement-controlled loading rate of 2 mm/min. The average compressive strength of at least three randomly selected specimen was determined and reported.

The Vicat apparatus was used to determine the setting times, including initial and final, in accordance with Chinese GB/T 1346-2011 (Test methods for water requirement of normal consistency, setting time and soundness of the Portland cement) [51]. The test involves measuring the penetration resistance of a cement sample under specified conditions to determine the time at which the slurry reaches defined stiffness levels. The fluidity of the cementitious material slurry was measured according to Chinese GB/T 8077-2012 (Methods for testing uniformity of concrete admixture) [52] using a standard truncated conical mold. The slurry was filled into the mold placed on a flat glass plate, scraped flush, and then the mold was lifted vertically. After the slurry had flowed for 30 s on the glass plate, its diameter was measured in three different directions and the results were averaged to determine the fluidity of cementitious materials.

The samples cured at ambient temperature showed a slight volume change. The original lengths of the samples stored at 95% humidity and 20 ± 2 °C were measured using a vernier caliper with a 0.01 mm accuracy and recorded as *L*_0_, while the lengths of samples cured at ambient temperature for 1 day, 3 days and 7 days were measured and recorded as *L*′. The ambient temperature drying shrinkage of the specimen was then calculated using the following equation.

(2) $$\varepsilon =\frac{L_{0}-L^{\prime }}{L_{0}}\times 100\%$$

Since the cementitious materials are frequently injected around fire zones to seal air leaks for gas management and fire prevention, they are required to survive at elevated temperatures. To evaluate the high temperature durability of the cementitious materials, 7-day-cured cementitious specimens with lengths recorded as *L_c_* were heated to 100, 200, 300 and 400 °C in a muffle furnace, held at each temperature for an hour, and the lengths of the heated samples were measured and recorded as *L_t_* after cooling to room temperature. The thermal shrinkage rate was calculated using the following equation. The samples were subsequently subjected to compressive strength tests.

(3) $$\varepsilon_{t}=\frac{L_{c}-L_{t}}{L_{c}}\times 100\%$$

The morphology of the specimens before and after XG modification were obtained using a Quanta 250 field-emission scanning electron microscope (FEI, Medford, MA, USA), and the functional groups of the specimens were measured with a VERTEX 80V Fourier transform infrared spectrometer (Brucker, Billerica, Massachusetts, USA) with a scanning range of 4000–400 cm^−1^.

## 3. Result and Discussion

### 3.1. Analysis of FA-Based Cementitious Material Preparation by Response Surface Methodology

Table 3 shows the parameters and responses for the 15-run design of FA-based cementitious material synthesis. There were 12 factorial tests and 3 central-point repeated tests. The response parameters are the 1-day, 3-day and 7-day compressive strengths.

Table 3 shows that the 1-day, 3-day and 7-day compressive strengths varied from 0.0971 MPa to 16.7625 MPa, 0.1398 MPa to 29.3150 MPa, and 0.7850 MPa to 33.9105 MPa, respectively. The regression models for each response variable as functions of the GBFS-to-precursor (FA + GBFS) ratio (*X*_1_), the dosage of the alkali-activator (*X*_2_) and the modulus of the alkali-activator (*X*_3_) are summarized in Table 4. The accuracy of the regression model was evaluated by the correlation coefficient (*R*^2^). It is obvious that the *R*^2^ for the three responses is obtained in the range of 0.9421–0.9788, and the values of adjusted *R*^2^ are also higher than 0.8300, indicating that the model has good accuracy when predicting values.

An analysis of variance (ANOVA) was conducted to assess the significance of the model, and the results are summarized in Table 5. The probability value (*p*-value) was used to consider the statistical significance of the model, and its significance level is 0.05 or 0.1. It is observed that the compressive strengths at 1-day, 3-day and 7-day curing ages are affected by the experimental factors, with the exception of the modulus of the alkali-activator. Since the *p*-values proposed by the GBFS-to-precursor ratio and the dosage of the alkali-activator are less than 0.05, they are significant parameters on compressive strength, and the dosage of the alkali-activator has the most influence on the compressive strength, followed by the GBFS-to-precursor ratio; the optimal GBFS-to-precursor ratio was 0.35 for the 1-day compressive strength. Moreover, in the regression model analysis with the compressive strengths as responses, the *p*-values of the interaction term of *X*_2_*X*_3_ (dosage of alkali-activator × modulus of the alkali-activator) are also smaller than 0.05, thus making important contributions to the models and indicating that these parameters should be considered simultaneously while preparing FA-based cementitious materials.

**Figure 3:**
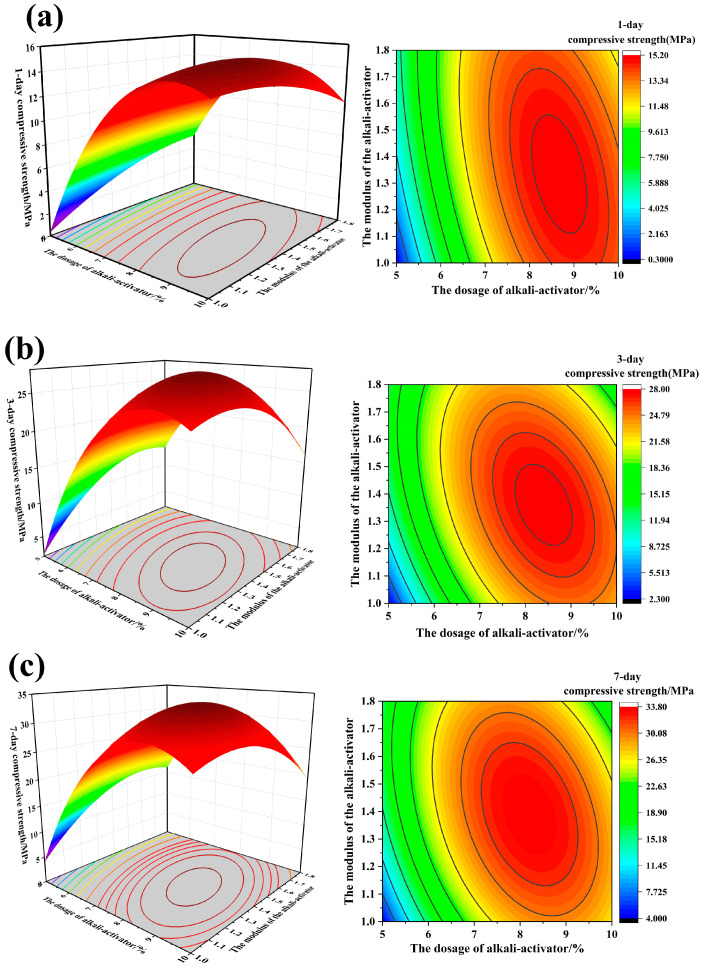
Response surface diagrams (**left**) and contour plots (**right**) of *X*_2_*X*_3_ on the compressive strength of FA-based cementitious material: (**a**) 1-day compressive strength; (**b**) 3-day compressive strength; (**c**) 7-day compressive strength.

In order to study the interactive effect of ***X***_2_***X***_3_ on the compressive strength of FA-based cementitious material, three-dimensional response surface diagrams and contour plots are plotted and presented in Figure 3.

According to Figure 3, the 1-day, 3-day and 7-day compressive strengths increase with an increasing alkali-activator dosage and alkali-activator modulus. As shown in the contour plots, when the modulus of the alkali-activator is low, there is an obvious increase in compressive strength with an increase in the alkali-activator dosage. This is because the modulus of the alkali-activator represents the molar ratio of SiO_2_ to Na_2_O, and a lower modulus indicates a higher alkali-activator dosage. With the increase in alkali-activator dosage, more precursors are depolymerized to generate more monomers such as SiO_4_^−^ and AlO_4_^5−^, and then polycondensation formed aluminosilicate gels, which increased the compressive strength. The contour plots showed that the optimal range for achieving the maximum compressive strength is an alkali-activator dosage between 7.5 and 9.5%, with an alkali-activator modulus of approximately 1.4. As a result, the optimum parameters for the synthesis of FA-based cementitious material in the formulation were as follows: GBFS-to-precursor ratio = 0.35, alkali-activator modulus = 1.4 and alkali-activator dosage = 7.5–9.5%.

### 3.2. Workability of FA-Based Cementitious Materials with Various Alkali-Activator

**Figure 4:**
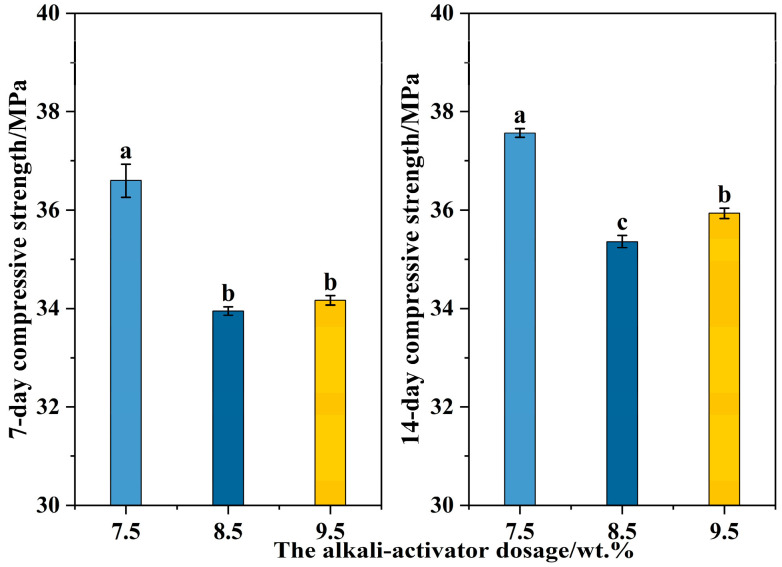
Compressive strength of FA-based cementitious material with different alkali-activator dosages. Different lowercase letters above the bars indicate statistically significant differences among groups (*p* < 0.05).

Under the optimal GBFS ratio (0.35) and the optimal alkali-activator modulus (1.4), three samples with alkali-activator dosages of 7.5%, 8.5% and 9.5% were prepared to obtain the optimal alkali-activator dosage for the preparation of the FA-based cementitious material. These three obtained samples were named FA-alkali-7.5, FA-alkali-8.5 and FA-alkali-9.5, respectively. Figure 4 presents a comparison of the specimens’ compressive strength after 7 and 14 days of curing. The compressive strength of the cementitious material decreased with an increase in the dosage of the alkali-activator, with the highest compressive strength observed in the sample activated by 7.5% alkali-activator. Furthermore, no statistically significant difference (indicated by the same lowercase letter, *p* < 0.05) in the 7-day compressive strength was found between the samples activated by 8.5% and 9.5% alkali-activator. This may be due to the fact that increasing the dosage of the alkali-activator accelerates the activation rate on the precursors’ surface, but when the alkali-activator is excessive, the rate of hydration products formed on the surface of the precursors is too fast, and the hydration products cannot be diffused, forming a protective film on the surface of the precursors’ particles, inhibiting the further activation of the substrate and resulting in a decline in compressive strength [53]. Therefore, the highest 7-day and 14-day compressive strengths of 36.60 MPa and 37.57 MPa, respectively, were achieved using a formulation with the following parameters: GBFS-to-precursor ratio = 0.35 (FA-to-precursor ratio = 0.65), alkali-activator modulus = 1.4 and alkali-activator dosage = 7.5%.

**Figure 5:**
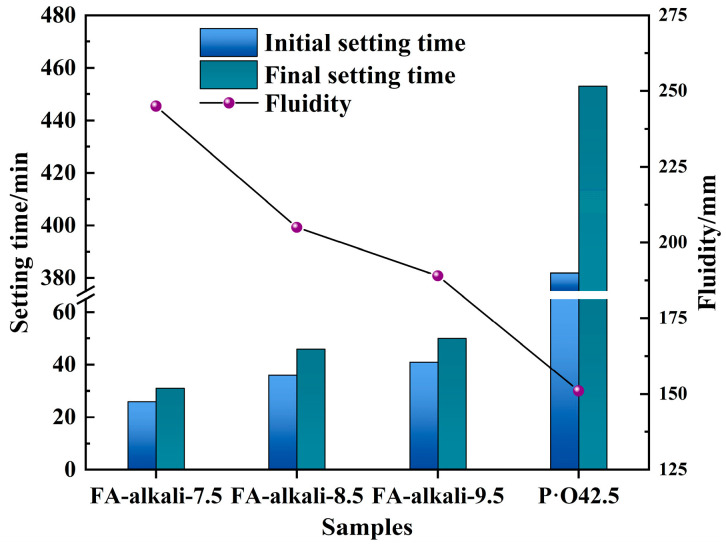
Setting time and fluidity of FA-based cementitious material with different alkali-activator dosages.

The setting time of the cementitious slurry is also a crucial factor in determining the coal mine sealing performance. Too quick coagulation of the sealing material slurry will result in tube blockage, preventing the slurry from reaching the desired location, whereas too slow coagulation would result in slurry loss after reaching the desired location, decreasing the sealing effect. The setting time of FA-based cementitious materials with various alkali-activator dosages was evaluated and compared to P·O42.5, and the results are summarized in Figure 5.

As illustrated in the figure, the setting times of FA-based cementitious materials are significantly shorter than those of P·O42.5 slurry. And with the increase in the alkali-activator dosage, the setting time of the FA-based cementitious materials increased while the fluidity decreased. This phenomenon can be attributed to excessive alkali-activator inhibiting the activation rate inside the precursor particles, which is consistent with the change in the compressive strength of samples activated with varied alkali-activator dosages. It was observed that FA-based cementitious material activated by 7.5% alkali-activator exhibited the shortest setting time and the highest fluidity, with initial and final setting times of 26 min and 31 min, respectively, and a fluidity of 245 mm.

In summary, the FA-based cementitious material activated by 7.5% alkali-activator can maintain the highest fluidity for about half an hour before condensing and achieving the highest compressive strength, which can fully meet the requirements of underground coal mine grouting for sealing leakage and preventing fire. Therefore, considering the compressive strength, setting time and fluidity, the optimum result for the synthesis of FA-based cementitious material is GBFS-to-precursor ratio = 0.35, alkali-activator modulus = 1.4 and alkali-activator dosage = 7.5%. The optimal FA-based cementitious material is referred to as Op-FG.

### 3.3. High-Temperature Resistance of Op-FG

**Figure 6:**
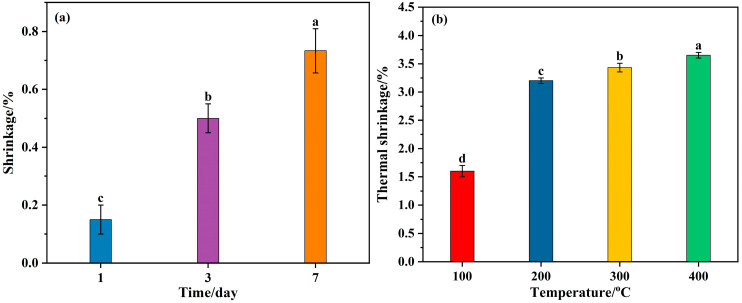
Shrinkage of Op-FG: (**a**) ambient temperature drying shrinkage over time and; (**b**) thermal shrinkage after exposure to heat. Different lowercase letters indicate statistically significant differences among groups (*p* < 0.05).

Generally, sealing materials are applied around the outer boundary of the fire area to block potential air leakage channels. Therefore, the fire prevention and sealing materials are required to possess certain high-temperature resistance, particularly in terms of anti-shrinkage and resistance to thermally induced cracking properties. The temperature outside the fire zone is typically not too high due to the low thermal conductivity of coal and rock. Therefore, this paper only focused on the high-temperature resistance of the cementitious sealing materials up to 400 °C. Shrinkage, including ambient temperature drying shrinkage over time and thermal shrinkage, are depicted in Figure 6.

Since the drying shrinkage of alkali-activated material was found to reach approximately 50% of the 91-day shrinkage after the first 7 days [54], indicating that the alkali-activated material had already undergone significant shrinkage at an early stage, the shrinkage of Op-FG at ambient temperature during the first 7 days was investigated. As observed in Figure 6a, due to the evaporation of free water in Op-FG, the sample gradually shrank, and the ambient drying shrinkage rate was 0.73% after curing for 7 days, which was significantly lower than the values for alkali-activated fly ash and ground granulated blast furnace slag reported by Cyr et al. [55]. However, the thermal shrinkage of Op-FG increased with the increase in heating temperature (Figure 6b), as the evaporation rate of free water was accelerated by heating, and the differences were statistically significant, as indicated by different lowercase letters (*p* < 0.05). The lowest thermal shrinkage was achieved after exposure to 100 °C, reaching 1.6%. When the heating temperature increased to 200 °C, the thermal shrinkage rate increased sharply to 3.2%, representing an increase rate of 100%. However, as the heating temperature increased further, the thermal shrinkage rate increased gradually and had little change. This may be attributed to the fact that the water present in the sample is predominately in the form of free water, which evaporates after heating at 100–200 °C, leading to sample shrinkage. Lahoti et al. also reported a similar thermal shrinkage phenomenon in metakaolin geopolymers; however, the shrinkage was observed within a range of 5.5–7.5% up to 300 °C [56], which is significantly higher than that of Op-FG.

**Figure 7:**
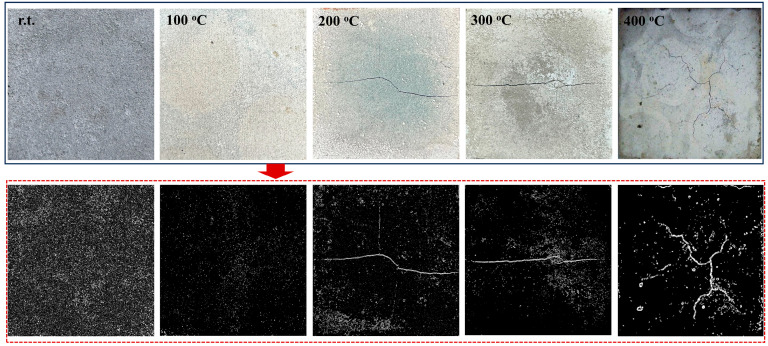
Raw optical and binary images of Op-FG before and after heating: r.t. represents before heating, and 100 ℃, 200 ℃, 300 ℃ and 400 ℃ represent after heating.

The crack patterns of the Op-FG samples before and after exposure to elevated temperatures were examined, and the representative images of heated samples are shown in Figure 7. All crack morphologies were analyzed using Image J 1.47. Raw optical images were first converted to 8-bit grayscale, and contrast enhancement was then applied uniformly across all images. Cracks were segmented using adaptive thresholding to account for non-uniform illumination and surface texture. The binary images obtained after adaptive thresholding were presented in the second row of Figure 7. It can be seen that there is no obvious crack on the surface of Op-FG after exposure to 100 °C, but the color of the sample becomes lighter, which may be due to iron oxidation in the fly ash [57]. However, as the heating temperature increased further, the free water within the matrix of the sample evaporates at an accelerated rate, leading to pore shrinkage and crack formation. Visible cracks were observed on the sample after exposure to 200 °C and higher temperatures, and the sample showed more damage after exposure to 300 °C compared to the sample after exposure to 200 °C. Furthermore, after exposure to 400 °C, the samples exhibited not only larger cracks but also the formation of multiple air bubbles.

Thus, thermal shrinkage and even cracking after heating still exist in Op-FG prepared under optimal reaction conditions despite its high compressive strength, appropriate setting time and fluidity, which makes it easy to form secondary air leakage and decreases the fire prevention effect. Therefore, there is still an urgent need to improve its thermal shrinkage and cracking resistance.

### 3.4. Xanthan Gum Modified Op-FG Cementitious Material

In order to improve the thermal shrinkage and cracking resistance of the FA-based cementitious sealing material, three batches of xanthan-gum-modified Op-FG cementitious materials were prepared by incorporating xanthan gum into the slurry of Op-FG with increasing xanthan gum contents (1‰, 2‰, and 3‰), as shown in Table 6.

**Figure 8:**
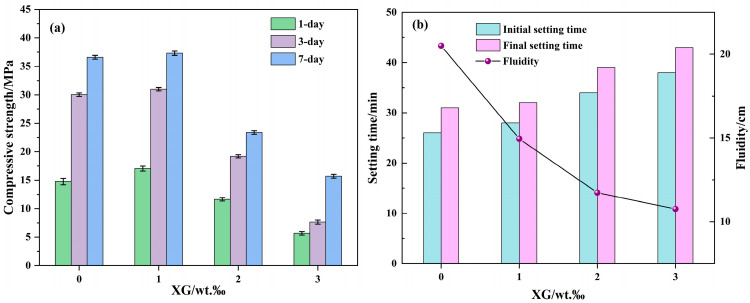
Compressive strengths (**a**) setting times and fluidity (**b**) of modified cementitious materials with different XG amounts.

The 1-day, 3-day, and 7-day compressive strengths of modified cementitious materials with varied XG amounts (1‰, 2‰, and 3‰) are shown in Figure 8a. For the reference sample (FG), the compressive strength was 14.76 MPa at 1 day, 30.04 MPa at 3 days, and increases to 36.59 MPa after 7 days of curing. When 1 wt.‰ XG was incorporated into the FG matrix, the compressive strength increased from 14.76 MPa to 17.04 MPa at 1 day, with an increase of 15.44%. It increased from 30.04 MPa to 30.99 MPa at 3 days and from 36.59 MPa to 37.31 MPa at 7 days, with increases of 3.16% and 1.97%, respectively. This is due to the fact that XG with branch chains can spontaneously form a polymer gel network and could also be cross-linked with Al^3+^ and Ca^2+^ to form hydrogels. The newly formed crosslinked network was interpenetrated with the original hydrated aluminosilicate gel (C(N)-A-S-H) network, thereby enhancing the structural stability of the cementitious material and increasing its compressive strength. However, further increasing the XG content to 2‰ and 3‰ led to a notable decline in compressive strength. The sample with 3‰ XG exhibited the lowest values, with 1-day, 3-day and 7-day compressive strengths of 5.66 MPa, 7.65 MPa and 15.71 MPa, respectively. This significant decrease in compressive strength can be attributed to the excess XG results in uneven mixing due to the viscosity of XG, forming more defects in the cementitious gel system and weakening the compressive strength of the material. Although the current results indicate that an excessive XG content negatively affects mechanical properties, several potential strategies could be explored in future studies to mitigate this strength loss. For example, the incorporation of nano-silica or nano-clay may compensate for the reduction in strength by providing additional nucleation sites and promoting microstructural densification. Combining XG with other biopolymers, such as cellulose nanofibers, to simultaneously achieve viscosity control and mechanical reinforcement may also prove beneficial.

The setting times and fluidity of the XG-modified materials provide further insight, as illustrated in Figure 8b. The addition of 1‰ XG had minimal effect on the initial and final setting times but resulted in a sharp decrease in fluidity compared to FG. With higher XG contents (2‰ and 3‰), the setting times were progressively extended, and the fluidity continued to decline. These observed trends were attributed to increased XG incorporation, which resulted in an increase in the viscosity of the cementing material slurry. Obviously, with an increase in XG incorporation, in addition to forming a gel network with Al^3+^ and Ca^2+^ in the matrix and weakening the fluidity of the slurry, excessive XG also increased the viscosity of the slurry, decreased the fluidity, and slowed the hydration reaction of the cementitious material, so that the setting time was prolonged. It is apparent that the 1‰ XG-modified FA-based cementitious material (XFG1) has a considerable setting time, like FG, but that the fluidity (15.0 cm) was reduced by 27.07% compared to FG. In general, the poor fluidity of cementitious sealing materials may lead to pipe blocking, whereas materials with appropriate fluidity can effectively fill air leakage channels and avoid the phenomenon of pipe blocking. However, excessive fluidity may result in slurry loss, thereby reducing the sealing efficiency. In our previous work, a cement sealing material that has been widely used in coal mine only had a fluidity of 11.3 cm, which increased the difficulty of grouting [41]. It should be noted that the 1‰ XG-modified FA-based cementitious material can set in about 30 min and achieve a compressive strength of 17 MPa after 1 day. It exhibits superior fluidity compared to conventional cement sealing materials, and the appropriate fluidity and setting time are conducive to the pumping and sealing of cementitious fire-fighting materials. Therefore, the 1‰ XG-modified FA-based cementitious material (XFG1) was prepared for further investigation.

### 3.5. High-Temperature Performance of XFG1

**Figure 9:**
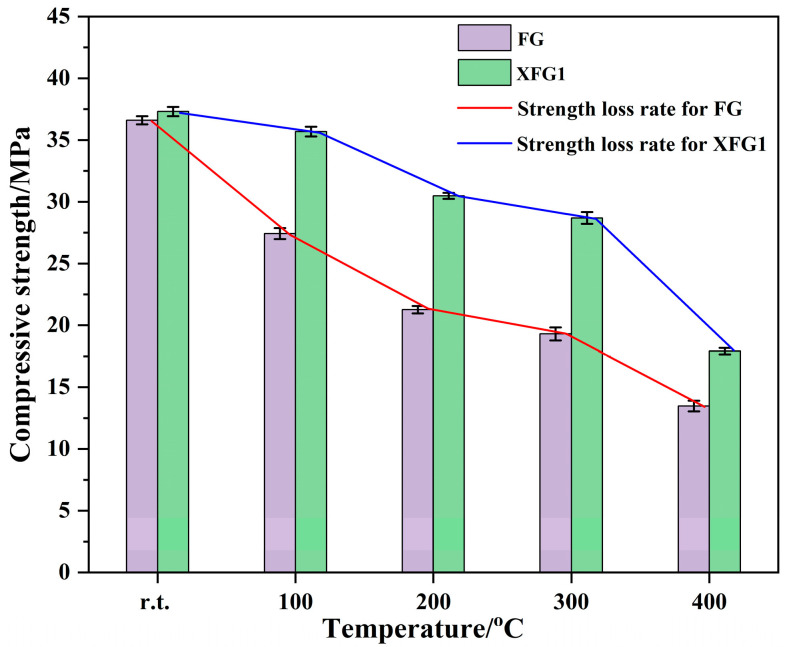
Compressive strength of FG and XFG1 after heating.

As discussed above, the incorporation of 1‰ XG could enhance the compressive strength and fluidity of FA-based cementitious sealing materials. However, can the incorporation of 1‰ XG also improve the high temperature resistance of FA-based cementitious materials? To address this question, the high-temperature resistance of XFG1, including residual compressive strength after heating, thermal shrinkage and thermal cracking, was evaluated and compared with that of FG. Given that the cementitious sealing materials are commonly injected around fire zones to seal air leakage pathways and prevent coal spontaneous combustion, and considering the geothermal gradient, the thermal properties of rocks, and the combined effects of multiple heat sources, this study specifically evaluates the high-temperature resistance performance of cementitious sealing materials under temperatures not exceeding 400 °C, in line with the relatively low temperature typically observed in the periphery of underground coal mine fire zones [41]. Figure 9 depicts the compressive strengths of FG and XFG1 after exposure to heat, with unheated samples serving as references. As shown in the figure, the compressive strength of both FG and XFG1 decreased after heating, and the residual compressive strength of the sample decreased gradually with the increase in heating temperature, which was mainly attributed to the structural damage caused by the crack formation of free water loss and the decomposition of cementitious matrix. It is worth noting that when heated at 100 °C and 200 °C, the compressive strength of FG decreased significantly faster than XFG1. The residual compressive strength for XFG1 specimens after exposure to 100 °C decreased from 37.3 MPa before heating to 35.6 MPa, with a decrease of only 4.5%, while the compressive strength of FG after heating at 100 °C decreased by 25.1%. When the heating temperature was increased to 200 °C, the compressive strength of FG decreased by 22.3% and that of XFG1 decreased by 14.6% compared to the residual compressive strengths of samples after exposure to 100 °C. It was observed that the residual compressive strengths of XFG1 were always higher than those of FG after heating, even when the heating temperature was increased to 300 °C, and that the compressive strength loss rate of XFG1 was similar to that of FG; when the heat temperature was raised to 400 °C, the compressive strength loss rate of XFG1 was greater than that of FG. Obviously, the incorporation of XG improved the residual compressive strength of FA-based cementitious material after exposure to high temperatures, which was probably attributed to the cross-linking network between XG and metal ions, which improves the stability of the gel.

**Figure 10:**
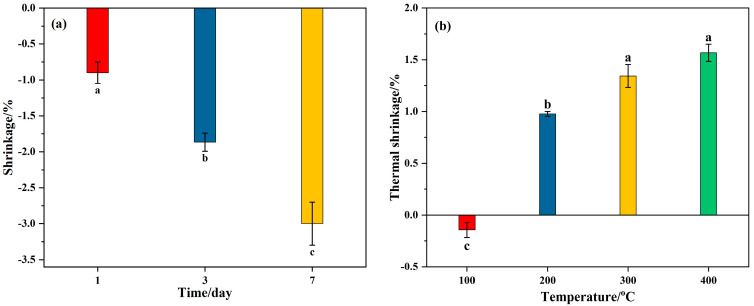
Shrinkage of XFG1: (**a**) ambient temperature drying shrinkage over time and; (**b**) thermal shrinkage after exposure to heat. Different lowercase letters indicate statistically significant differences among groups (*p* < 0.05).

The ambient-temperature drying shrinkage over time and thermal shrinkage for XFG1 were also studied, and the results are depicted in Figure 10. To our surprise, XFG1 showed a slight expansion at ambient temperature, and the expansion rate gradually increased with time (Figure 10a). This expansion phenomenon may be due to the swelling properties of the crosslinked gel generated by XG and metal ions (Al^3+^/Ca^2+^) in the matrix, which expands after absorbing water during the standard curing process. On the other hand, unlike Portland cement, alkali-activated cementitious materials have been reported to have a large amount of unbound or free water that can evaporate to form cracks [58], which has been demonstrated in the study of shrinkage and crack development for Op-FG above. After the incorporation of XG into Op-FG, the hydrogel formed by crosslinking XG with Al^3+^/Ca^2+^ in the gelling system absorbed the free water in the matrix and swelled, thereby preventing the evaporation of free water, resulting in sample shrinkage.

Figure 10b showed the thermal shrinkage of XFG1 after exposure to 100–400 °C. Unlike Op-FG, XFG1 expanded slightly after exposure to 100 °C instead of shrinking. This may be due to the fact that the crosslinking reaction between XG and Al^3+^/Ca^2+^ accelerated at 100 °C and more hydrogels were formed. However, when the heating temperature was increased to 200–400 °C, the XFG1 specimen also inevitably showed shrinkage due to the water loss of crosslinked hydrogel after heating at high temperatures, and the thermal shrinkage also increased with the increase in temperature. Meanwhile, there is no significant difference in the thermal shrinkage rate between 300 °C and 400 °C, which may be because the dehydrated crosslinked hydrogel filled in the void of the gel and increased the stability of the structure of the cementitious material. Although XFG1 also showed shrinkage after being heated at 200–400 °C, it should be noted that the thermal shrinkages of Op-FG are higher than 3% at within this temperature range (Figure 6a), whereas the thermal shrinkages of XFG1 are much lower than that of FG, reaching only 1.5% even after heating at 400 °C, indicating that XG could improve the thermal shrinkage resistance of FA-based cementitious materials.

**Figure 11:**
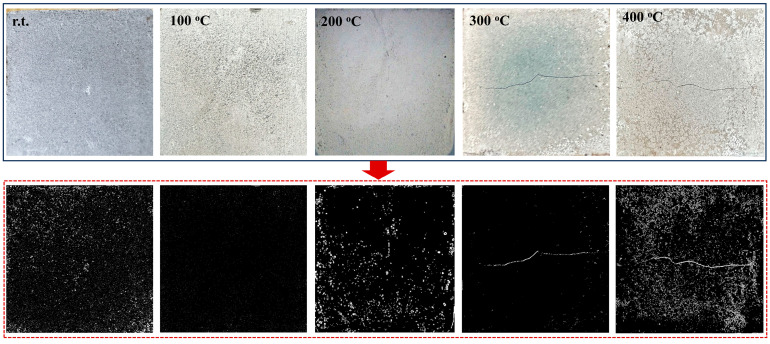
Raw optical and binary images of XFG1 before and after heating.

Figure 11 showed the crack development of XFG1 samples before and after exposure to elevated temperatures. Different from Op-FG, no cracks were observed on the surface of XFG1 after being heated at 100 and 200 °C. Moreover, although cracks did appear on the surface of XFG1 when the temperature was increased to 300 and 400 °C, the extent of damage was significantly less compared to that of Op-FG under the same conditions. This is primarily due to the fact that the hydrogel formed through the crosslinking of XG with Al^3+^/Ca^2+^ possesses both water absorption and water retention capabilities, which can effectively delay the cracking caused by water evaporation from the matrix of the cementitious material, thereby improving its structural stability. This observation is consistent with the residual compressive strength and shrinkage observations related to the structural stability of the cementitious materials.

In summary, the high-temperature resistance, including residual compressive strength, thermal shrinkage resistance and cracking resistance, can be significantly improved by the incorporation of 1‰ XG into FA-based cementitious material.

### 3.6. Microstructural Characterization

The microstructure of FG and XFG1 was analyzed by FTIR and SEM, and the effect of XG on the structure of FA-based cementitious material was discussed.

**Figure 12:**
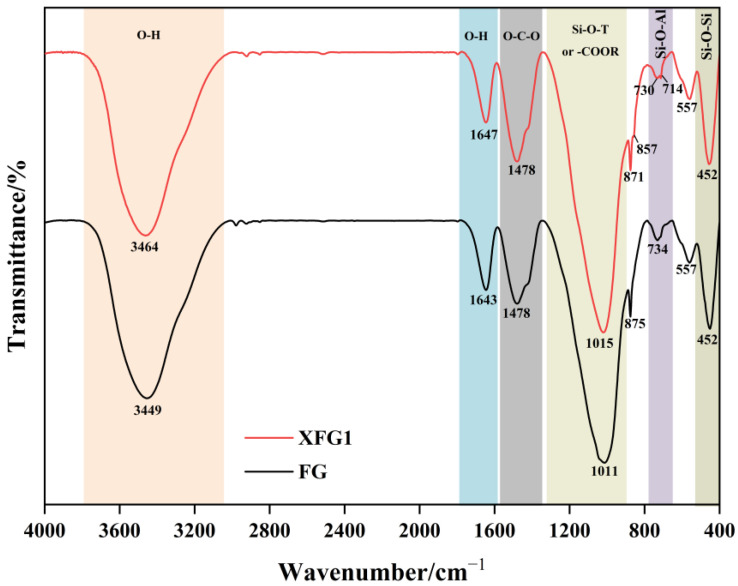
FTIR spectra of FG and XFG1 specimens.

A comparison of FTIR spectra for FG and XFG1 samples at 7 days is shown in Figure 12. In general, the infrared spectra of FG and XFG1 showed great similarity, with the main absorption peaks as well as the corresponding functional groups being identified as follows. The adsorption bands around 3460 cm^−1^ and 1650 cm^−1^ are assigned to stretching vibrations of -OH and bending vibrations of H-O-H for free water and bound water in the cementitious samples [59,60]. The stretching vibration peaks of O-C-O are located at 1478 cm^−1^, indicating the presence of CO_3_^2−^, which may be produced by carbonation of the cementitious materials [61]. The bending vibration peaks located at 730 cm^−1^ and 452 cm^−1^ are assigned to Si-O-Al and Si-O-Si, respectively [60].

**Figure 13:**
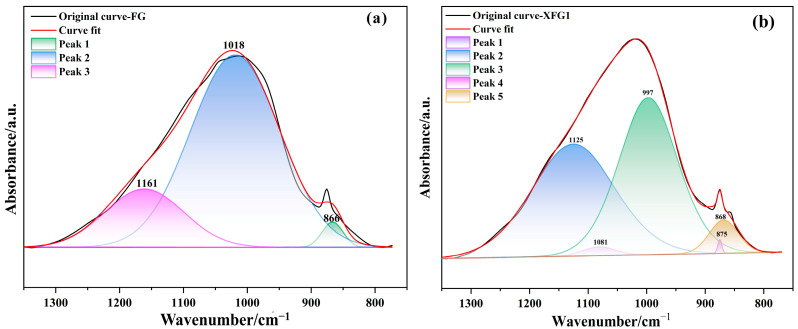
Deconvoluted spectra of (**a**) FG; and (**b**) XFG1 (in the range of 1330–800 cm^−1^.

A broad band (1330–800 cm^−1^) appeared in both FG and XFG1, which is attributed to the stretching vibrations of Si-O-T (T is Si or Al) or carboxylate. Peak separation, which can identify the different peaks and bands, was applied on this broad band of infrared spectra to compare the microstructure of FG and XFG1. Figure 13 showed the deconvoluted bands in the range of 1330–800 cm^−1^ for FG and XFG1.

As shown in Figure 13a, the bands at approximately 1160 cm^−1^ and 1018 cm^−1^ are assigned to Q^4^ and Q^3^ silicon tetrahedra in the silica gel, indicating that the structure of FA-based cementitious material (FG) is predominantly composed of a silica gel framework. Meanwhile, the little peak that appeared at ~860 cm^−1^ is attributed to the C-O stretching vibration associated with carbonation in the cementitious material [62]. Due to the incorporation of XG, the bands corresponding to Q^4^ and Q^3^ silicon tetrahedra shifted towards lower wavenumbers of 1125 cm^−1^ and 997 cm^−1^, respectively, indicating an increase in the degree of polymerization after modification by XG. It should also be emphasized that, apart from these two peaks, an additional characteristic peak was observed at 1081 cm^−1^ in the FTIR spectrum of XFG1, which may be attributed to the asymmetric stretching vibration of carboxylate groups. Furthermore, in the infrared spectrum of XFG1, a characteristic peak at 875 cm^−1^ assigned to the *β*-glycosidic linkages of XG was observed alongside the carbonate adsorption peak at 868 cm^−1^. This clearly indicates that XG was successfully incorporated into the FA-based cementitious matrix, thereby enhancing the degree of polymerization in the gelling system.

**Figure 14:**
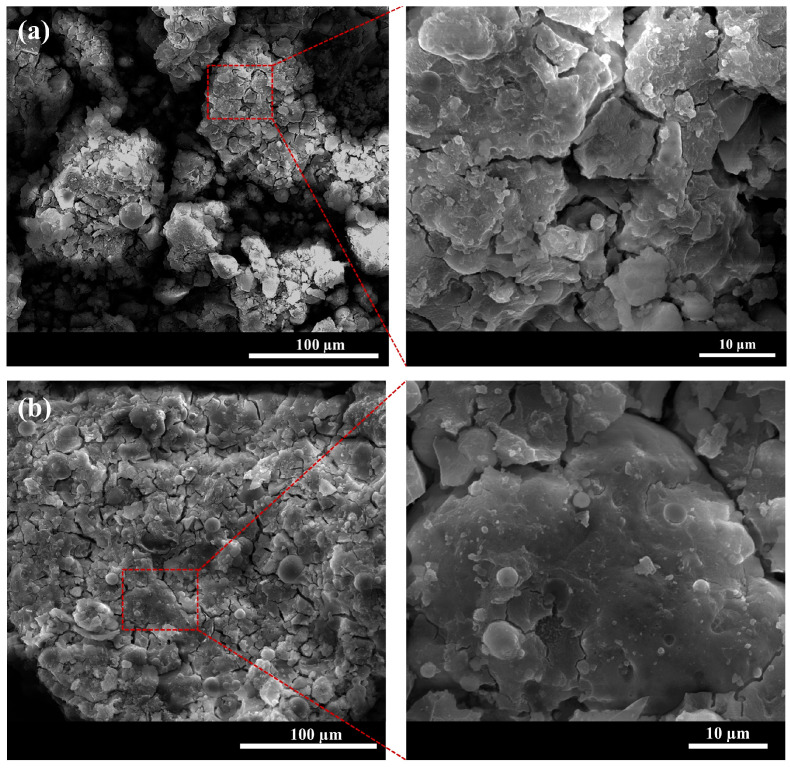
SEM images of FG (**a**) and XFG1; (**b**) specimens.

SEM analysis provides further insight into the modification of XG on the structure of the FA-based cementitious material. The SEM images of FG and XFG1 specimens were presented in Figure 14. As shown in the figure, the surface of FG appears loose, with an incomplete matrix structure and the presence of flocculent products (Figure 14a). In contrast, when XG was added, the matrix’s structure became more compact, and polymerization resulted in a more complete network, leading to improved homogeneity and a smoother, denser morphology (Figure 14b). This enhancement can be attributed to the following two reasons: first, the hydroxyl group in XG crosslinks with Al^3+^/Ca^2+^ to generate hydrogels, strengthening the stability of the original gelling structure; on the other hand, XG exhibits a certain viscosity, and a small amount of XG in the gelling system can increase the interfacial adhesion of fine aggregates.

### 3.7. Mechanism Analysis of Xanthan Gum Modified FA-Based Cementitious Material

**Figure 15:**
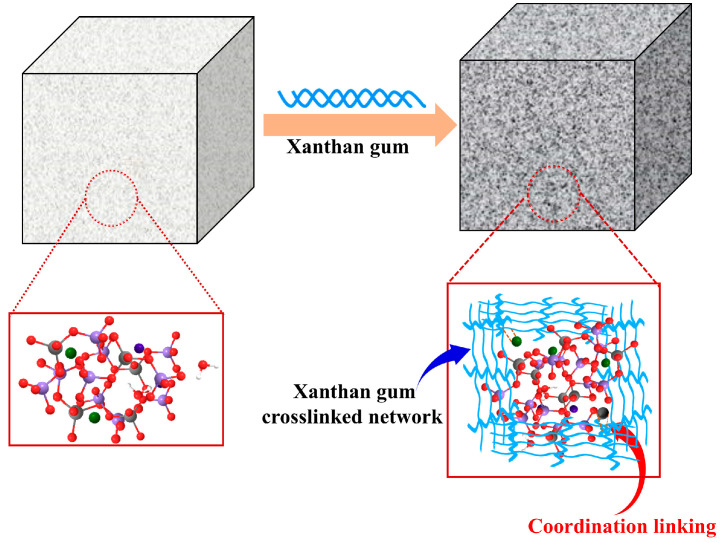
Modification of XG on FA-based cementitious material.

Fly ash and ground granulated blast furnace slag are the main precursors of the xanthan-gum-modified FA-based cementitious material prepared in this study. It has been reported that the formation of alkali-activated FA-based cementitious material underwent several processes of dissolution, condensation to form silicate oligomers and polycondensation to form gels, during which Si-O-Si, Al-O-Al and Si-O-Al covalent bonds were broken to form active monomers, and then polymerized to form hydrated aluminosilicate gels, resulting in the formation of the three-dimensional network structure of the gelled material [27]. When XG was added to the matrix of FA-based cementitious material, some modification occurred (Figure 15). Firstly, XG with a structure of branch chains has been reported to easily form a dense and homogeneous polymer gel network that strengthens the structural stability of the original hydrated aluminosilicate gel [63]. Secondly, Al^3+^ and Ca^2+^ present in the FA-based gelling matrix would coordinate with the carboxyl groups of XG chains to form a coordination crosslinked gel, thereby increasing the crosslinking degree of the system [62,63,64,65,66]. The highly crosslinked gels formed by XG are interspersed and bonded in the C(N)-A-S-H gel network to form a double gel network, which not only increases the compressive strength of the cementitious material, but also enhances the stability of the gel structure, thus improving the crack resistance at high temperatures. Meanwhile, the hydrogel generated by XG features water absorption and water retention properties. These properties make the modified FA-based cementitious material not only avoid shrinkage, but also exhibit slight expansion. Additionally, the water retention property helps to slow down the cementitious material’s shrinkage and cracking caused by water loss during heating, thereby preventing the formation of secondary air leakage channels after the fire.

However, it should be noted that although XG could improve the high-temperature resistance and compressive strength of FA-based cementitious materials, this modifying effect does not increase with the amount of XG. In other words, the increase in XG is unfavorable regarding the modification of the cementitious material. As demonstrated in Figure 8, with an increase in XG content, the compressive strength of the XG-modified cementitious material decreased, the setting time was prolonged, and the fluidity decreased. This behavior can be attributed not only to the increased viscosity caused by XG, but also to the gel structure formed by XG. It has been found that Al^3+^ coordinates preferentially with the terminal carboxyl group of XG, and Zeng et al. calculated the relative energies of geometric configurations of the coordination of Al^3+^ with the terminal carboxyl groups and non-terminal carboxyl groups of XG, and the results confirmed this [67]. Some Al^3+^ ions were generated during the formation of alkali-activated cementitious material. Al^3+^ preferentially coordinated with terminal carboxyl groups, while excess Al^3+^ coordinated with non-terminal carboxyl groups. However, the coordination pattern with non-terminal carboxyl groups was found to break down the gel network and generate flocculation, thus decreasing the structural stability [67]. This is also the reason why the compressive strength of XG-modified FA-based cementitious material decreased with the increase in XG. Therefore, considering the structure stability and fluidity of the modified gel, only 1‰ XG modification is required.

## 4. Conclusions

In this work, an alkali-activated coal-fly-ash-based mining cementitious sealing material mix design was developed using response surface methodology. The regression models described the functional relationship between the compressive strength (1-day, 3-day and 7-day compressive strength) and three variables: the GBFS-to-precursor (FA+GBFS) ratio, the dosage of alkali-activator and the modulus of the alkali-activator. Under the optimized conditions of a 0.35 GBFS-to-precursor ratio, 1.4 alkali-activator modulus, and 7.5% alkali-activator dosage, the initial and final setting times approached 26 min and 31 min, respectively, the fluidity was 245 mm, and the 7-day compressive strength approached 36.60 MPa; however, thermal shrinkage and cracking after heating still exist in the FA-based cementitious material prepared under optimal reaction conditions.

Due to its viscosity and coordination crosslinking with metal ions, xanthan gum (XG) was then introduced to improve the thermal shrinkage and cracking of FA-based cementitious materials. Incorporating 1‰ XG decreased the fluidity while maintaining the setting time and increased the 1-day and 7-day compressive strength by 15.44% and 1.97%, respectively. In general, 1‰ XG-modified FA-based cementitious material also showed minor expansion at ambient temperatures and improved residual compressive strength, thermal shrinkage and cracking in comparison to FG. However, due to the viscosity of XG and the coordination of Al^3+^ and non-terminal carboxyl groups in XG breaking the gel network, XG incorporation should not exceed 1‰, as the compressive strength and fluidity were decreased.

The microstructural study involving FTIR and SEM revealed that XG was successfully incorporated into an FA-based cementitious matrix, increasing the polymerization of the gelling system and the interfacial adhesion of fine aggregates. The gels generated by XG cross-linking and coordinating with Al^3+^/Ca^2+^ not only have the property of water absorption and swelling, which delays the water loss of the cementitious material, but they are also interspersed in the original C(N)-A-S-H gel network, resulting in a stable structure with improved resistance to thermal cracking and shrinkage.

In summary, XG-modified FA-based cementitious material with improved thermal cracking and shrinkage resistance realizes the resource utilization of coal fly ash. It is a promising cementitious material for coal mine fire prevention with low carbon emissions that could replace cement-based mining sealing materials.

## Figures and Tables

**Table 1:** The oxide composition (wt.%) of FA and GBFS [42].

Metal Oxides	Oxide Composition (wt.%)	
	FA	GBFS
SiO_2_	47.2	28.2
Al_2_O_3_	41.6	15.1
CaO	2.3	36.8
Fe_2_O_3_	4.0	0.3
MgO	0.5	8.5
TiO_2_	1.4	1.3
Na_2_O	0.5	0.5
K_2_O	0.9	0.4
Other ^a^	1.6	8.9

^a^ Other metal oxides with low abundance.

**Table 2:** Levels of each factor for synthesis of FA-based cementitious material.

Factors	Low Level	Medium Level	High Level
Factor 1: GBFS-to-precursors (FA + GBFS) ratio	0.25	0.30	0.35
Factor 2: the dosage of alkali-activator (wt.%)	5.0%	7.5%	10.0%
Factor 3: the modulus of the alkali-activator	1.0	1.4	1.8

**Table 3:** Experimental design of FA-based cementitious material.

Run	Experimental Factors			Response Variables		
	*X*_1_	*X*_2_	*X*_3_	*Y*_1_/MPa	*Y*_2_/MPa	*Y*_3_/MPa
1	0.3	5%	1	0.0971	0.1398	0.7850
2	0.25	7.50%	1	9.6183	18.1667	23.0600
3	0.35	7.50%	1	14.4958	29.3150	33.9105
4	0.3	10%	1	12.4792	21.3650	23.9963
5	0.25	5%	1.4	1.2854	5.6350	8.1663
6	0.35	5%	1.4	3.2592	17.3263	19.6725
7	0.3	7.50%	1.4	13.1900	24.8188	32.1206
8	0.3	7.50%	1.4	14.4500	30.3850	32.2706
9	0.3	7.50%	1.4	14.7000	25.5525	34.2844
10	0.25	10%	1.4	8.8300	19.9300	20.2969
11	0.35	10%	1.4	16.7625	27.1413	31.8538
12	0.3	5%	1.8	6.6417	15.2325	18.8081
13	0.25	7.50%	1.8	8.9783	15.0875	19.0406
14	0.35	7.50%	1.8	14.3375	26.9125	30.6244
15	0.3	10%	1.8	11.1850	18.2338	22.4119

**Table 4:** Statistical data and the equation for each response in terms of coded factors.

Response	*R* ^2^	Adjusted *R*^2^	Regression Model Equation
Compressive strength			
1-day	0.9788	0.9405	$Y_{1}=14.11+2.52X_{1}+4.75X_{2}+0.5565X_{3}+1.49X_{1}X_{2}+0.1204X_{1}X_{3}-1.96X_{2}X_{3}-1.16X_{1}^{2}-5.42X_{2}^{2}-1.09X_{3}^{2}$
3-day	0.9421	0.8380	$Y_{2}=26.92+5.23X_{1}+6.04X_{2}+0.81X_{3}-1.12X_{1}X_{2}+0.1692X_{1}X_{3}-4.56X_{3}-0.3915X_{1}^{2}-9.02X_{2}^{2}-4.16X_{3}^{2}$
7-day	0.9436	0.8421	$Y_{3}=32.89+5.69X_{1}+6.39X_{2}+1.14X_{3}+0.0127X_{1}X_{2}+0.1833X_{1}X_{3}-4.90X_{2}X_{3}-1.37X_{1}^{2}-11.53X_{2}^{2}-4.87X_{3}^{2}$

**Table 5:** ANOVA results for the established regression model.

Source	*p*-Value		
	*Y*_1_	*Y*_2_	*Y*_3_
Model	0.0012	0.013	0.0122
***X***_1_	0.0025	0.0075	0.0087
***X***_2_	0.0001	0.0041	0.0054
***X***_3_	0.2691	0.5323	0.4402
***X***_1_ ***X***_2_	0.0654	0.5411	0.995
***X***_1_ ***X***_3_	0.8567	0.925	0.9279
***X***_2_ ***X***_3_	0.027	0.0445	0.0516
***X***_1_^2^	0.1384	0.8345	0.5254
***X***_2_^2^	0.0004	0.0039	0.0022
***X***_3_^2^	0.1577	0.0666	0.0597
Lack of Fit	0.2347	0.4309	0.0584

**Table 6:** Composition of XG modified Op-FG cementitious materials.

Series	Composition
FG	Op-FG cementitious materials
XFG1	Op-FG + 1 wt.‰ XG
XFG2	Op-FG + 2 wt.‰ XG
XFG3	Op-FG + 3 wt.‰ XG

## Data Availability

The original contributions presented in this study are included in the article material. Further inquiries can be directed to the corresponding author.
